# The role of exercise in restoring executive function: a comparison of tobacco-exposed college athletes and sedentary students

**DOI:** 10.3389/fphys.2024.1499587

**Published:** 2024-12-02

**Authors:** Minjia Wang, Shuya Wu, Qian Ma, Hao Hu, Yanpei Liu, Yaozheng Wang, Shitao Zhan, Dongsen Liu, Olivier Girard

**Affiliations:** ^1^ School of Sport Medicine and Health, Chengdu Sport University, Chengdu, China; ^2^ The School of Sports Medicine and Rehabilitation, Beijing Sport University, Beijing, China; ^3^ School of Human Sciences, Exercise and Sport Science, University of Western Australia, Perth, WA, Australia

**Keywords:** exercise, sedentary individuals, executive function, smoking, prefrontal cortex

## Abstract

**Introduction:**

As adolescent smoking rates rise, its impact on cognitive function has drawn greater attention. This study explores whether exercise can mitigate the negative effects of smoking on executive function in male college students.

**Methods:**

Sixty male college students were divided into four groups (n = 15 each): sedentary smokers, sedentary nonsmokers, athletic smokers, and athletic nonsmokers. All participants completed the Eriksen flanker task, with prefrontal cortex activation measured using functional near-infrared spectroscopy. After the baseline test, all sedentary students engaged in 33 min of high-intensity interval training, followed by the same procedures as in the pre-test.

**Results:**

In the flanker task, college athletes exhibited superior executive function compared to sedentary students, with higher accuracy (*p* = 0.042), faster reaction times (*p* = 0.002), and more pronounced brain activation (*p* = 0.048). Post-exercise, reaction times improved significantly in sedentary groups (*p* < 0.05). Smoking impaired executive function both before and after exercise, with smokers showing lower accuracy (*p* < 0.001), slower reaction times (*p* < 0.001), and diminished brain activation (*p* < 0.001) compared to nonsmokers.

**Discussion:**

Engaging in acute aerobic exercise may improve executive function in sedentary smokers. Exercise may help mitigate smoking-related declines in executive function among college students.

## Introduction

Smoking is a major public health issue, contributing to over 8 million deaths annually, according to World Health Organization in 2019 (Caulfield and Garrett, 2002). It has well-documented harmful effects on the respiratory, cardiovascular, and immune systems, contributing to 10% of all cardiovascular diseases and nearly 90% of deaths from lung cancer and chronic obstructive pulmonary disease (Dahdah et al., 2022). Smoking is particularly prevalent in China, home to over one-third of the world’s male smokers (Chen et al., 2015; Li et al., 2016). In recent years, smoking rates have risen among younger populations. The 2018 China-Global Adult Tobacco Survey (GATS) revealed that the average age for starting daily smoking is 21.1 years, aligning with the typical age of Chinese college students. Additionally, smoking behavior significantly increases during college compared to high school (Xie et al., 2022).

Research increasingly focuses on the effects of smoking on cognitive function. A 2011 study from the Population Aging Knowledge Base of India (BKPAI) found that older adults who smoked were 24% more likely to experience cognitive impairment compared to non-smokers (Muhammad et al., 2021). A study of middle-aged white individuals with at least 5 years of smoking history revealed consistent underperformance in various cognitive domains including cognitive efficiency and executive skills. Adolescent smokers also show significant cognitive deficits. A UK longitudinal study of individuals aged 13–18 years linked adolescent tobacco use to impairments in working memory, response inhibition, and emotional recognition (Mahedy et al., 2021; Chang et al., 2021). Additionally, adults aged 18–29 assessed with the Cambridge Neuropsychological Test Automated Battery (CANTAB) exhibited significant deficits in sustained attention, spatial cognitive, and executive planning compared to non-smokers (Chamberlain et al., 2012). Overall, smoking is consistently associated with poorer cognitive outcomes across different age groups and cognitive domains (Durazzo et al., 2012). However, the interaction between smoking and other lifestyle factors remains largely unexplored.

Exercise, as an active lifestyle, contrasts with smoking by potentially increasing brain volume and improving executive function (Colcombe et al., 2006). Studies indicate that individuals with long-term exercise habits, such as athletes, generally have superior cognitive function compared to their sedentary counterparts (Schott and Krull, 2019; Tseng et al., 2013; Yu et al., 2021). For instance, master athletes possess higher concentrations of gray and white matter in brain regions associated with visuospatial function, motor control, and working memory compared to similarly aged and educated sedentary adults (Tseng et al., 2013). In young adults, exercise activates the prefrontal cortex, thereby improving cognitive inhibitory control (Byun et al., 2014; Zhang et al., 2021). Athletes also tend to outperform sedentary individuals in tasks involving behavioral inhibition and error monitoring (Yu et al., 2021). A study on college students revealed that acute exercise improves cognitive function in healthy young adults, with high-intensity exercise leading to greater cognitive gains than moderate-intensity or no exercise (Ballester-Ferrer et al., 2022). However, it remains unclear whether exercise can mitigate the negative effects of smoking on cognitive function.

Research on the cognitive effects of smoking traditionally used questionnaires, such as CANTAB (Al-Mshari et al., 2020) and the Bayley Scales of Infant Development III (BSID-III) (Horton et al., 2020). However, recent studies have shifted from relying on cognitive scales (Chamberlain et al., 2012; Durazzo et al., 2012; Morales et al., 2014) to employing brain imaging techniques such as functional near-infrared spectroscopy (fNIRS), function magnetic resonance imaging (fMRI), and electroencephalography (EEG) to examine the effects of exercise on cognition and monitor brain hemodynamic changes. Compared to EEG, fNIRS offers better spatial resolution (approximately 2.5–3 cm) and can image up to 1–2 cm in depth (McCormick et al., 1992), making it highly effective for cortical areas (Hu et al., 2020). Additionally, fNIRS is more portable, easier to use in various environments, and less prone to motion artifacts than fMRI (Hu et al., 2020).

This study aimed to use fNIRS to investigate the effects of acute exercise on executive function in sedentary college students, both smokers and nonsmokers, and compare these effects with those in college athletes. Our intention is to determine whether long-term exercise habits could mitigate the negative impact of smoking. We hypothesized that sedentary smokers would show more pronounced improvements in executive function following an acute exercise session compared to their baseline levels.

## Material and methods

### Participants

The sample size was estimated using G*Power 3.1 software, with an effect size of 0.25, alpha of 0.05, and power of 0.8, indicating a minimum requirement of 40 participants. Sixty individuals aged 18–30 were recruited from Chengdu Institute of Physical Education (China) and divided into four groups: college athletic smokers (n = 15), college athletic nonsmokers (n = 15), college sedentary smokers (n = 15), and college sedentary nonsmokers (n = 15). Inclusion criteria were i) right-handedness, ii) no neurological diseases, and iii) no prior participation in cognitive function experiments. Athletes had over 2 years of regular sports training and were classified at Tier 2 (trained/developmental) skill level (McKay et al., 2022). Sedentary participants did not meet the criteria of 30 min of moderate-intensity physical activity 5 days a week, 20 min of vigorous-intensity physical activity 3 days a week, or an equivalent of 600 metabolic equivalent (MET)-minutes per week (Hallal et al., 2012). Smokers were defined as those smoking at least 10 cigarettes per day for the past 6 months with a Fagerstrom Test for Nicotine Dependence (FTND) score of ≥6. Non-smokers had smoked fewer than 100 cigarettes in their lifetime and had not smoked in the past 30 days (Newland et al., 2019). Exclusion criteria followed the American College of Sports Medicine guidelines, with pre-exercise screening conducted using the 2022 PAR-Q+ questionnaire (The Physical Activity Readiness Questionnaire for Everyone; see supplementary material). The study was approved by the Ethics Committee of Chengdu Institute of Physical Education and Sports (approval number: [2023] 146) and registered under ChiCTR2300076123. All procedures adhered to the Helsinki declaration, and participants provided informed consent.

### Procedure

**FIGURE 1:**
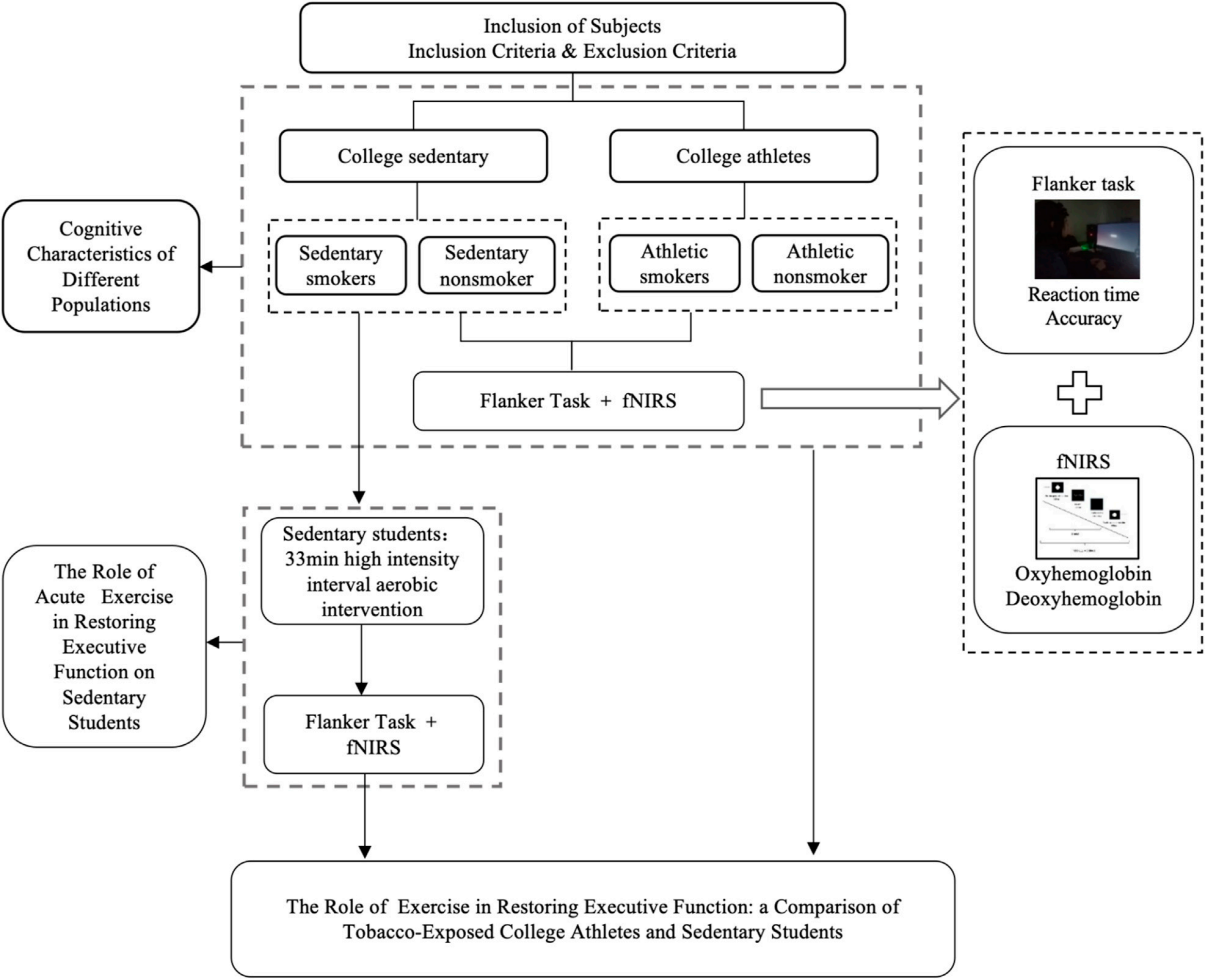
Experimental flow chart.

This study employed a single-blind design, maintaining anonymity through identification codes. The experiment consisted of three phases: pre-intervention, intervention, and post-intervention (Figure 1). During the pre-intervention phase, participants were briefed, signed informed consent form, supplied basic information, completed the 2022 PAR-Q+, and took the flanker test.

**FIGURE 2:**
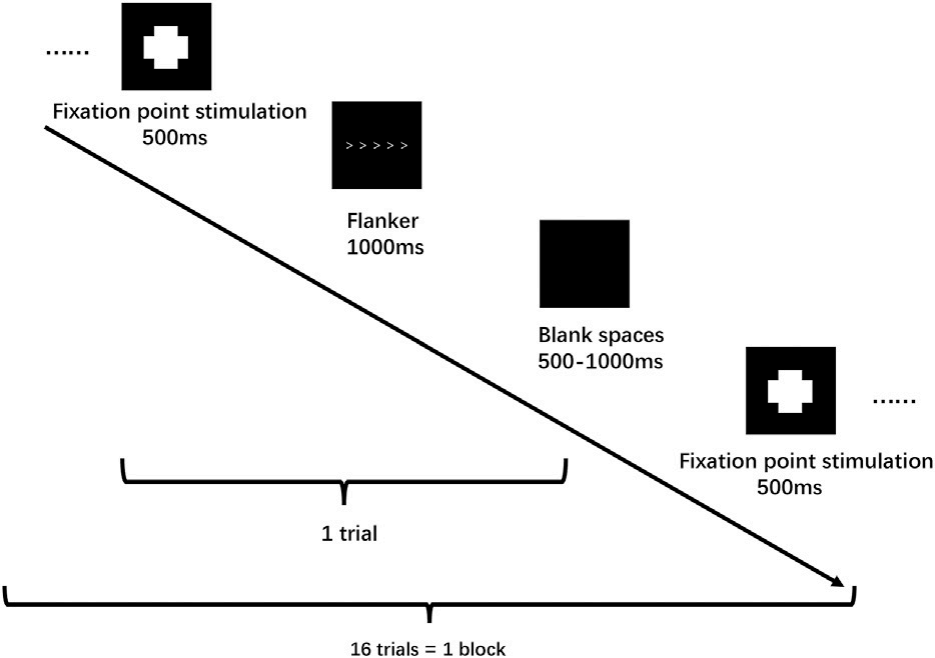
Design of the Flanker mission block.

All participants were required to refrain from consuming beverages containing caffeine or alcohol and to avoid any vigorous physical activity for 24 h prior to testing. Participants wore fNIRS devices for monitoring prefrontal cortex (PFC) hemoglobin oxygen (oxy-Hb) concentration while simultaneously completing the flanker task, both before and after the intervention. They first put on the fNIRS cap and adjusted the light source and detector probe. The task was performed in a dimly lit room. Participants were instructed: *“For the next 13 min, sit comfortably, do nothing other than tap on the keyboard, do not talk or fall asleep, and complete the flanker task paradigm.”* The flanker task comprised a 6-trial practice session followed by 96 formal trials. Stimuli consisted of five arrows, with congruent stimuli and incongruent stimuli (Figure 2). Participants judged the direction of the central arrow and pressed the corresponding key. After each response, the target marker reappeared, and the next trial began. A 60-s rest period was provided between each block.

**FIGURE 3:**
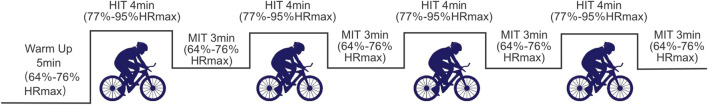
Acute aerobic intervention flowchart.

After the pre-intervention test, the two sedentary groups completed 33 min of high-intensity interval aerobic exercise on a power bicycle ergometer under supervision at the Cardiopulmonary Laboratory at Chengdu Institute of Physical Education. The protocol included a 5-min warm-up at 64%–76% of maximal heart rate, followed by 4 min of high-intensity aerobic exercise at 77%–95% of maximal heart rate, and 3 min of recovery at 64%–76% of maximal heart rate (Liguori, 2018). Heart rate was monitored using the Polar H10 Heart Rate monitor (Kempele, Finland), which participants wore across their chest, with maximal heart rate calculated as (220-age) × 100% (Liguori, 2018). This cycle was repeated four times, totaling 33 min (Figure 3).

To avoid acute exercise-induced changes in skin blood flow having an effect on fNIRS measurements, a 15-min rest was required before performing the fNIRS test under the flanker task again (Wu et al., 2024). Post-intervention testing used the same procedures as the pre-test. The athlete groups, serving as controls, completed only the pre-intervention phase.

### Study outcomes

#### Flanker task

The Eriksen flanker task was used to measure selective attention and inhibitory function in the neuroscientific experiment (Kałamała et al., 2018), assessing both reaction time and accuracy. Participants completed the task while seated and stationary during fNIRS measurement to ensure accurate capture of brain activity related to working memory and sensory functions, minimizing interference from body movements.

#### Oxyhemoglobin saturation

A multi-channel NIRSport2 portable near-infrared imager (NIRX, United States) was used to monitor cerebral blood oxygen signals. The imager sampled at 10.2 Hz using two infrared wavelengths (760 nm and 850 nm) and featured eight light sources and seven detectors, totaling 20 channels spaced 3 cm apart. The setup, arranged according to the international 10–20 system, enabled precise capture of hemodynamic changes in prefrontal brain regions during the flanker task.

The fNIRS data were preprocessed using nirsLAB software. Optical density data were band-pass filtered (0.01–0.3 Hz) and converted to oxy-Hb values using the modified Beer-Lambert law. Data were processed using the HRF function and input into the GLM model to extract oxy-Hb beta values. Since oxy-Hb is more sensitive to exercise effects than deoxygenated hemoglobin (Pinti et al., 2020), these values were used to assess cortical oxygenation and neural activity in the PFC.

### Statistical analysis

The oxy-Hb signals were processed using nirs LAB software. Behavioral data and processed Beta values were collated using Excel 2016, with statistical analyses conducted using SPSS 26.0 software. Continuous data, which were normally distributed and had homogeneous variance (checked using chi-square), are presented as mean ± standard deviation (‾X ± SD). One-way ANOVA was used to compare the baseline differences among the four groups. Behavioral outcomes (reaction time and accuracy) and oxy-Hb concentrations in the PFC during congruent and incongruent tasks, pre-intervention, were analyzed using two-way ANOVA: smoking habits (smoking and nonsmoking) × exercise habits (athletic and sedentary). Additionally, behavioral outcomes and oxy-Hb concentrations were analyzed using a two-way ANOVA with the factors of smoking habits (sedentary smoking group and sedentary nonsmoking group) × interventions (pre-intervention and post-intervention). Bonferroni corrected *post hoc* tests were followed. Partial eta-squared (η^2^) was calculated as a measure of effect size, with values of 0.01, 0.06 and >0.14 considered *small*, *medium* and *large*, respectively. Statistical significance was set at *p* < 0.05.

## Results

### Sample characteristic

Demographics of participants are depicted in Table 1. No significant differences were found among the four groups.

**TABLE 1:** Anthropometric characteristics of participants (M ± SD).

	Athletic smokers (n = 15)	Athletic nonsmokers (n = 15)	Sedentary smokers (n = 15)	Sedentary nonsmokers (n = 15)	F (3,56)	*P*-value
Age (y)	22.60 ± 2.13	22.40 ± 1.50	22.53 ± 1.36	22.60 ± 1.84	0.044	0.987
Weight (kg)	71.57 ± 8.16	69.41 ± 5.77	69.66 ± 5.96	69.80 ± 9.60	0.257	0.856
Height (m)	1.74 ± 0.05	1.76 ± 0.04	1.75 ± 0.04	1.75 ± 0.05	0.223	0.880
BMI (kg/m^2^)	23.57 ± 2.59	22.50 ± 1.55	22.77 ± 1.35	22.77 ± 2.60	0.723	0.542

College participants were divided into four groups (n = 15 in each group): athletic smokers, athletic nonsmokers, sedentary smokers, and sedentary nonsmokers.

### Flanker task

#### Accuracy

**FIGURE 4:**
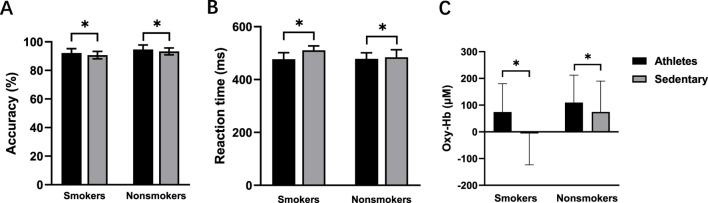
Accuracy **(A)**, reaction time **(B)**, and oxy-Hb signals **(C)** for the athletes and sedentary groups during the incongruent task. *p < 0.05 denotes a significant difference between the athletes and sedentary groups.

**FIGURE 5:**
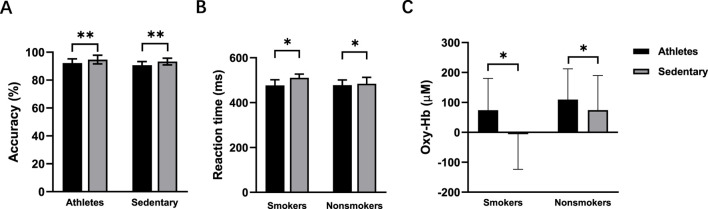
Accuracy **(A)**, reaction time **(B)**, and oxy-Hb signals **(C)** for the smokers and nonsmokers during the incongruent task. *p < 0.05 denotes a significant difference between smokers and nonsmokers. **p < 0.001 denotes a significant difference between smokers and nonsmokers.

There was a significant main effect of exercise habit on both congruent (F (1, 56) = 4.164, *p* = 0.046, η2 = 0.069) and incongruent tasks (F (1, 56) = 4.331, *p* = 0.042, η2 = 0.072) (Figure 4A), with athletes showing significantly higher accuracy rates than sedentary individuals. For the incongruent task, smoking habit had a significant main effect (F (1,56) = 12.333, *p* = 0.001, η2 = 0.180), indicating lower accuracy in smokers (Figure 5A). However, there was no significant main effect of smoking habit (*p* = 0.077) for the congruent task.

**FIGURE 6:**
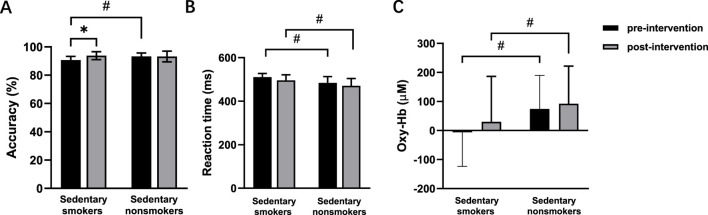
Accuracy **(A)**, reaction time **(B)**, and oxy-Hb signals **(C)** pre- and post-intervention in sedentary smokers (n = 15) and sedentary nonsmokers (n = 15) during the incongruent task. *p < 0.05 denotes a significant difference between pre- and post-intervention. #*p* < 0.05 denotes a significant difference between sedentary smokers and sedentary nonsmokers in the pre-intervention.

During the congruent task, neither exercise habit (*p* = 0.106) nor time (*p* = 0.352) had significant main effects in the post-intervention test. For the incongruent task, there were no main effects of time (*p* = 0.065) or smoking habit (*p* = 0.065). However, there was a significant interaction effect between time and smoking habit (F (1,56) = 4.367, *p* = 0.041, η2 = 0.072) (Figure 6A). Simple effects analysis revealed a significant increase in accuracy for smokers after the intervention (*p* = 0.007) (Table 2).

**TABLE 2:** The accuracy, reaction time and oxy-Hb signal of the Flanker congruent task (M ± SD).

Index	Athletic Smokers (n = 15)	Athletic nonsmokers (n = 15)	Sedentary Smokers (n = 15)		Sedentary nonsmokers (n = 15)	
	Pre-intervention		Pre-intervention	Post-intervention	Pre-intervention	Post-intervention
Congruent accuracy (100%)	98.5 ± 2.3	99.0 ± 13.4	96.7 ± 3.4	97.9 ± 1.8	98.3 ± 1.2	98.2 ± 1.5
Incongruent accuracy (100%)	92.2 ± 3.0	94.7 ± 3.1	90.7 ± 2.6	93.8 ± 2.8*	93.3 ± 2.4	93.1 ± 3.8
Congruent reaction times (ms)	407.9 ± 25.7	394.4 ± 25.9	409.4 ± 28.5	404.8 ± 14.0*	404.4 ± 17.4	380.4 ± 17.0*
Incongruent reaction times (ms)	476.8 ± 25.0	478.1 ± 22.9	511.0 ± 16.2	495.7 ± 25.4*	484.2 ± 28.6	470.6 ± 33.7*
Congruent Oxy-Hb signal (μM)	6.27 ± 139.02	44.90 ± 130.08	18.51 ± 138.16	57.15 ± 119.35	14.03 ± 112.18	47.60 ± 113.72
Incongruent Oxy-Hb signal (μM)	74.13 ± 106.07	109.47 ± 102.90	6.20 ± 117.43	29.80 ± 156.73	74.39 ± 115.37	92.16 ± 129.70

* *p* < 0.05 denotes a significant difference between pre- and post-intervention.

#### Reaction time

Pre-intervention, neither exercise habit (*p* = 0.373) nor smoking habit (*p* = 0.152) had significant main effects in the congruent task, and there was no interaction effect between exercise and smoking habits (*p* = 0.514). In the incongruent task, there were significant main effects for both exercise (F (1, 56) = 10.937, *p* = 0.002, η2 = 0.163) (Figure 4B) and smoking habit (F (1, 56) = 4.341, *p* = 0.042, η2 = 0.072) (Figure 5B), along with a significant interaction effect between exercise and smoking habits (F (1, 56) = 5.300, *p* = 0.025, η2 = 0.086). College sedentary smokers had significantly longer reaction times compared to college athletic smokers (*p* < 0.001), while college athletic nonsmokers had significantly faster reaction time compared to college athletic smokers (*p* = 0.003).

In the congruent task, there was a main effect of time (F (1, 56) = 6.875, *p* = 0.011, η2 = 0.109), indicating a decrease in reaction time post-intervention. There was also a main effect of smoking habit (F (1, 56) = 9.560, *p* = 0.003, η2 = 0.146), showing that nonsmokers had faster reaction times. In the incongruent task, there were main effects of time (F (1, 56) = 4.394, *p* = 0.041, η2 = 0.073) and smoking habit (F (1,56) = 14.105, *p* < 0.001, η2 = 0.201) (Figure 6B).

#### Functional near-infrared spectroscopy (fNIRS)

Before the intervention, no significant differences were found between smokers and nonsmokers (*p* = 0.295) or between athletes and sedentary individuals (*p* = 0.412) in the congruent condition. In the incongruent condition, there were significant main effects of exercise (F (1, 56) = 4.077, *p* = 0.048, η2 = 0.068; Figure 4C) and smoking habit (F (1, 56) = 4.114, *p* = 0.047, η2 = 0.068; Figure 5C), with higher oxy-Hb signals in nonsmokers and athletes (Table 2).

The main effect of time was non-significant in both the congruent task (*p* = 0.087) and the incongruent task (*p* = 0.430) (Figure 6C). There was no main effect of smoking habit (*p* = 0.715) in the congruent task. However, the incongruent task showed a significant main effect of smoking habit (F (1,56) = 4.471, *p* = 0.039, η2 = 0.074), with higher oxy-Hb signals in nonsmokers. There were no significant interaction effects between time and smoking habit in either the congruent task (*p* = 0.054) or incongruent task (*p* = 0.789).

## Discussion

To examine how smoking and exercise habits interact to affect executive functioning in young adults, we recruited college athletes and sedentary students, categorizing them into four groups based on smoked status. All participants completed the flanker task while their Oxy-Hb signals were monitored with fNIRS. Key findings include: i) both smoking and a sedentary lifestyle impair executive function; ii) acute aerobic exercise enhances executive function in smokers. These findings support our hypothesis that college sedentary smokers experience greater improvements in executive function after acute intense exercise.

### Effects of smoking on executive functions

Before the acute motor intervention, we examined the effects of smoking on executive function and brain activation in college athletes and sedentary students. Smokers showed lower accuracy and slower reaction times on the flanker task, indicating impaired executive function. Ernst et al. (2001) reported comparable findings with longer reaction times in smokers compared to nonsmokers and ex-smokers using the n-back task. Luijten et al. (2011) tested smokers and nonsmokers using the Go/No Go task and analyzed N2 signals measured by EEG. They found that smokers typically performed less accurately and had decreased No Go N2 amplitude, indicating impaired response inhibition. These findings are consistent with our observations of impaired executive function in smokers.

We also found that nonsmokers had more pronounced PFC brain activation during the flanker incongruent task compared to smokers. This suggests that tobacco smoke may suppress activation in brain regions related to cognitive function, such as the frontal lobe. Consistent with this, diffusion tensor imaging revealed increased diffusivity in white matter bundles of the frontal lobe among smokers, indicating potential abnormalities (Gruber and Yurgelun-Todd, 2005). These impairments may be related to oxidative stress from smoking. Manna et al. (2006) found increased reactive oxygen species and lipid peroxidation in the frontal cortex of mice exposed to cigarette smoke, suggesting chronic exposure leads to tissue damage. Darnai et al. (2019) used fMRI to demonstrate that smoking disrupts connections between the arachnoid nucleus and the prefrontal cortex, impairing judgment, decision-making, and impulse control. Our study did not explore the impact of smoking on functional connectivity between brain regions, which should be addressed in future research.

### Effects of exercise on executive functions

Prior to the intervention, college athletes showed superior executive control compared to sedentary students. Athletes with long-term exercise habits exhibited more pronounced brain activation, faster reaction times, and higher accuracy during incongruent tasks compared to sedentary students. These differences are likely attributable to lifestyle habits (i.e., sedentary *versus* active). Loprinzi and Kane (2015) have investigated the effects of sedentary on cognitive functioning in healthy young adults and found that it was negatively correlated with visual attention and task-switching performance. Conversely, long-term exercise enhances executive function by increasing blood flow and cortical activity, which improves information processing and attention, and inhibitory control (Chan et al., 2022). Our findings show that among college students who smoke, those with regular exercise habits showed faster reaction times. This suggests that long-term exercise can mitigate some of the negative effects of smoking on cognitive function. A study demonstrated that a year-long aerobic exercise intervention improved cognitive function by increasing cerebral blood flow perfusion and reducing cerebrovascular stiffness (Tomoto et al., 2023). Monitoring cerebral blood flow before and after 12 weeks of exercise with a transcranial Doppler flow analyzer (EMS-9WA) identified cerebral blood flow as a potential mechanism for exercise-induced improvements in executive function in young individuals (Liu et al., 2023). Our results support these findings, as increased cerebral blood flow during the flanker task, measured by fNIRS, was associated with enhanced executive function.

We observed significant improvements in accuracy and reaction time in the sedentary smoking group after acute exercise. However, oxygen levels and brain activation remained significantly higher in nonsmokers, suggesting that while acute exercise can improve executive function in smokers, it does not fully counteract the negative effects of smoking. These findings align with previous research showing that exercise generally benefits executive function and inhibitory control in adolescents (Liang et al., 2022). The observed benefits of acute exercise may be attributed to increased production of brain-derived neurotrophic factor (BDNF) (Wheeler et al., 2020), which supports neurons and helps reverse learning and memory deficits (Liu et al., 2022). However, the extent of these benefits may vary based on an individual’s physical fitness. For instance, Hogan et al. (2013) used EEG to show that adolescents with higher fitness levels had significantly faster reaction time post-exercise, while those with lower fitness levels did not, implying that higher fitness is associated with better cortical efficiency. Since our study involved only sedentary college students undergoing the acute intervention, further research is needed to explore how intervention effects might differ among individuals with varying levels of athleticism.

This study has some limitations. Firstly, while cognitive functions involve complex brain networks (Ardila and Ostrosky, 2022), the fNIRS equipment only measured the frontal lobe, limiting our ability to explore functional connections with other brain areas (e.g., the supplementary motor area). Secondly, the benefits of exercise on executive function may differ based on physical fitness levels, leaving the effectiveness of interventions between high- and low-level athletes unexplored. Thirdly, the study included only male college students, which may limit the generalizability of the findings to other populations, as the effect of smoking on executive function could vary by sex (Sabia et al., 2012). Additionally, we observed significant variability in individual blood oxygen data, a common phenomenon noted in comparable studies (Wu et al., 2024; Yao et al., 2022). It can not be excluded that larger sample sizes may reduce this variability and improve the robustness of our findings.

## Conclusion

This study aimed to investigate the effects of an acute exercise intervention on executive functioning in sedentary college students, and to determine whether long-term exercise habits can mitigate the negative effects of smoking. Our results indicated that both college athletes and sedentary smokers showed reduced executive functions. Nonetheless, 33 min of acute aerobic exercise effectively improved executive functions in smokers. While both smoking and a sedentary lifestyle negatively impact executive functions in college individuals, acute aerobic exercise proves to be an effective intervention to enhance executive functions diminished by smoking.

## Data Availability

The datasets presented in this article are not readily available. Requests to access the datasets should be directed to dongsam1001@sina.com.

## References

[B1] Al-Mshari A. A. S. AlSheikh M. H. Latif R. Mumtaz S. (2020). The effect of smoking on cognition as measured by Cambridge Neuropsychological Test Automated Battery (CATNAB) and brain-derived neurotrophic factor plasma levels. Saudi Med. J. 41 (12), 1308–1314. 10.15537/smj.2020.12.25513 33294889 PMC7841597

[B2] Ardila A. Ostrosky F. (2022). What do neuropsychological tests assess? Appl. Neuropsychol. Adult. 29 (1), 1–9. 10.1080/23279095.2019.1699099 31826667

[B3] Ballester-Ferrer J. A. Bonete-López B. Roldan A. Cervelló E. Pastor D. (2022). Effect of acute exercise intensity on cognitive inhibition and well-being: role of lactate and BDNF polymorphism in the dose-response relationship. Front. Psychol. 13, 1057475. 10.3389/fpsyg.2022.1057475 36570982 PMC9780502

[B4] Byun K. Hyodo K. Suwabe K. Ochi G. Sakairi Y. Kato M. (2014). Positive effect of acute mild exercise on executive function via arousal-related prefrontal activations: an fNIRS study. Neuroimage 98, 336–345. 10.1016/j.neuroimage.2014.04.067 24799137

[B5] Caulfield B. Garrett M. (2002). Functional instability of the ankle: differences in patterns of ankle and knee movement prior to and post landing in a single leg jump. Int. J. Sports Med. 23 (1), 64–68. 10.1055/s-2002-19272 11774069

[B6] Chamberlain S. R. Odlaug B. L. Schreiber L. R. Grant J. E. (2012). Association between tobacco smoking and cognitive functioning in young adults. Am. J. Addict. 21 (Suppl. 1), S14–S19. 10.1111/j.1521-0391.2012.00290.x 23786505

[B7] Chan Y. S. Jang J. T. Ho C. S. (2022). Effects of physical exercise on children with attention deficit hyperactivity disorder. Biomed. J. 45 (2), 265–270. 10.1016/j.bj.2021.11.011 34856393 PMC9250090

[B8] Chang K. Khandpur N. Neri D. Touvier M. Huybrechts I. Millett C. (2021). Association between childhood consumption of ultraprocessed food and adiposity trajectories in the avon longitudinal study of parents and children birth cohort. JAMA Pediatr. 175 (9), e211573. 10.1001/jamapediatrics.2021.1573 34125152 PMC8424476

[B9] Chen Z. Peto R. Zhou M. Iona A. Smith M. Yang L. (2015). Contrasting male and female trends in tobacco-attributed mortality in China: evidence from successive nationwide prospective cohort studies. Lancet 386 (10002), 1447–1456. 10.1016/S0140-6736(15)00340-2 26466050 PMC4691901

[B10] Colcombe S. J. Erickson K. I. Scalf P. E. Kim J. S. Prakash R. McAuley E. (2006). Aerobic exercise training increases brain volume in aging humans. J. Gerontol. A Biol. Sci. Med. Sci. 61 (11), 1166–1170. 10.1093/gerona/61.11.1166 17167157

[B11] Dahdah A. Jaggers R. M. Sreejit G. Johnson J. Kanuri B. Murphy A. J. (2022). Immunological insights into cigarette smoking-induced cardiovascular disease risk. Cells 11 (20), 3190. 10.3390/cells11203190 36291057 PMC9600209

[B12] Darnai G. Perlaki G. Zsidó A. N. Inhóf O. Orsi G. Horváth R. (2019). Internet addiction and functional brain networks: task-related fMRI study. Sci. Rep. 9 (1), 15777. 10.1038/s41598-019-52296-1 31673061 PMC6823489

[B13] Durazzo T. C. Meyerhoff D. J. Nixon S. J. (2012). A comprehensive assessment of neurocognition in middle-aged chronic cigarette smokers. Drug Alcohol Depend. 122 (1-2), 105–111. 10.1016/j.drugalcdep.2011.09.019 21992872 PMC3258460

[B14] Ernst M. Heishman S. J. Spurgeon L. London E. D. (2001). Smoking history and nicotine effects on cognitive performance. Neuropsychopharmacology 25 (3), 313–319. 10.1016/S0893-133X(01)00257-3 11522460

[B16] Gruber S. A. Yurgelun-Todd D. A. (2005). Neuroimaging of marijuana smokers during inhibitory processing: a pilot investigation. Brain Res. Cogn. 23 (1), 107–118. 10.1016/j.cogbrainres.2005.02.016 15795138

[B17] Hallal P. C. Andersen L. B. Bull F. C. Guthold R. Haskell W. Ekelund U. (2012). Global physical activity levels: surveillance progress, pitfalls, and prospects. Lancet. 380 (9838), 247–257. 10.1016/S0140-6736(12)60646-1 22818937

[B18] Hogan M. Kiefer M. Kubesch S. Collins P. Kilmartin L. Brosnan M. (2013). The interactive effects of physical fitness and acute aerobic exercise on electrophysiological coherence and cognitive performance in adolescents. Exp. Brain Res. 229 (1), 85–96. 10.1007/s00221-013-3595-0 23743717

[B19] Horton M. K. Zheng L. Williams A. Doucette J. T. Svensson K. Cory-Slechta D. (2020). Using the delayed spatial alternation task to assess environmentally associated changes in working memory in very young children. Neurotoxicology 77, 71–79. 10.1016/j.neuro.2019.12.009 31857145 PMC10129050

[B20] Hu X. Xiao G. Zhu K. Hu S. Chen J. I. U. Yu Y. U. N. (2020). Application of functional near-infrared spectroscopy in neurological diseases: epilepsy, stroke and Parkinson. J. Mech. Med. Biol. 20 (10), 2040023. 10.1142/s0219519420400230

[B21] Kałamała P. Szewczyk J. Senderecka M. Wodniecka Z. (2018). Flanker task with equiprobable congruent and incongruent conditions does not elicit the conflict N2. Psychophysiology 55 (2). 10.1111/psyp.12980 28845513

[B22] Li S. Meng L. Chiolero A. Ma C. Xi B. (2016). Trends in smoking prevalence and attributable mortality in China, 1991-2011. Prev. Med. 93, 82–87. 10.1016/j.ypmed.2016.09.027 27677441 PMC5124560

[B23] Liang X. Li R. Wong S. H. S. Sum R. K. W. Wang P. Yang B. (2022). The effects of exercise interventions on executive functions in children and adolescents with autism spectrum disorder: a systematic review and meta-analysis. Sports Med. 52 (1), 75–88. 10.1007/s40279-021-01545-3 34468951

[B15] Liguori G. (2018). ACSM’s guidelines for exercise testing and prescription. Headquarters in Amsterdam, Netherlands: Wolters Kluwer.

[B24] Liu J. Min L. Liu R. Zhang X. Wu M. Di Q. (2023). The effect of exercise on cerebral blood flow and executive function among young adults: a double-blinded randomized controlled trial. Sci. Rep. 13 (1), 8269. 10.1038/s41598-023-33063-9 37217511 PMC10203129

[B25] Liu S. Fan M. Xu J. X. Yang L. J. Qi C. C. Xia Q. R. (2022). Exosomes derived from bone-marrow mesenchymal stem cells alleviate cognitive decline in AD-like mice by improving BDNF-related neuropathology. J. Neuroinflammation 19 (1), 35. 10.1186/s12974-022-02393-2 35130907 PMC8822863

[B26] Loprinzi P. D. Kane C. J. (2015). Exercise and cognitive function: a randomized controlled trial examining acute exercise and free-living physical activity and sedentary effects. Mayo Clin. Proc. 90 (4), 450–460. 10.1016/j.mayocp.2014.12.023 25746399

[B27] Luijten M. Littel M. Franken I. H. (2011). Deficits in inhibitory control in smokers during a Go/NoGo task: an investigation using event-related brain potentials. PLoS One 6 (4), e18898. 10.1371/journal.pone.0018898 21526125 PMC3081309

[B28] Mahedy L. Wootton R. Suddell S. Skirrow C. Field M. Heron J. (2021). Testing the association between tobacco and cannabis use and cognitive functioning: findings from an observational and Mendelian randomization study. Drug Alcohol Depend. 221, 108591. 10.1016/j.drugalcdep.2021.108591 33618197 PMC8047806

[B29] Manna S. K. Rangasamy T. Wise K. Sarkar S. Shishodia S. Biswal S. (2006). Long term environmental tobacco smoke activates nuclear transcription factor-kappa B, activator protein-1, and stress responsive kinases in mouse brain. Biochem. Pharmacol. 71 (11), 1602–1609. 10.1016/j.bcp.2006.02.014 16569398 PMC2730355

[B30] McCormick P. W. Stewart M. Lewis G. Dujovny M. Ausman J. I. (1992). Intracerebral penetration of infrared light. Technical note. J. Neurosurg. 76 (2), 315–318. 10.3171/jns.1992.76.2.0315 1730963

[B31] McKay A. K. A. Stellingwerff T. Smith E. S. Martin D. T. Mujika I. Goosey-Tolfrey V. L. (2022). Defining training and performance caliber: a participant classification framework. Int. J. Sports Physiol. Perform. 17 (2), 317–331. 10.1123/ijspp.2021-0451 34965513

[B32] Morales A. M. Ghahremani D. Kohno M. Hellemann G. S. London E. D. (2014). Cigarette exposure, dependence, and craving are related to insula thickness in young adult smokers. Neuropsychopharmacology 39 (8), 1816–1822. 10.1038/npp.2014.48 24584328 PMC4059909

[B33] Muhammad T. Govindu M. Srivastava S. (2021). Relationship between chewing tobacco, smoking, consuming alcohol and cognitive impairment among older adults in India: a cross-sectional study. BMC Geriatr. 21 (1), 85. 10.1186/s12877-021-02027-x 33514331 PMC7847155

[B34] Newland N. Lowe F. J. Camacho O. M. McEwan M. Gale N. Ebajemito J. (2019). Evaluating the effects of switching from cigarette smoking to using a heated tobacco product on health effect indicators in healthy subjects: study protocol for a randomized controlled trial. Intern Emerg. Med. 14 (6), 885–898. 10.1007/s11739-019-02090-8 31049783 PMC6722146

[B35] Pinti P. Tachtsidis I. Hamilton A. Hirsch J. Aichelburg C. Gilbert S. (2020). The present and future use of functional near-infrared spectroscopy (fNIRS) for cognitive neuroscience. Ann. N. Y. Acad. Sci. 1464 (1), 5–29. 10.1111/nyas.13948 30085354 PMC6367070

[B36] Sabia S. Elbaz A. Dugravot A. Head J. Shipley M. Hagger-Johnson G. (2012). Impact of smoking on cognitive decline in early old age: the Whitehall II cohort study. Arch. Gen. Psychiatry 69 (6), 627–635. 10.1001/archgenpsychiatry.2011.2016 22309970 PMC3675806

[B37] Schott N. Krull K. (2019). Stability of lifestyle behavior - the answer to successful cognitive aging? A comparison of nuns, monks, master athletes and non-active older adults. Front. Psychol. 10, 1347. 10.3389/fpsyg.2019.01347 31231291 PMC6567482

[B38] Tomoto T. Verma A. Kostroske K. Tarumi T. Patel N. R. Pasha E. P. (2023). One-year aerobic exercise increases cerebral blood flow in cognitively normal older adults. J. Cereb. Blood Flow. Metab. 43 (3), 404–418. 10.1177/0271678X221133861 36250505 PMC9941859

[B39] Tseng B. Y. Uh J. Rossetti H. C. Cullum C. M. Diaz-Arrastia R. F. Levine B. D. (2013). Masters athletes exhibit larger regional brain volume and better cognitive performance than sedentary older adults. J. Magn. Reson Imaging 38 (5), 1169–1176. 10.1002/jmri.24085 23908143 PMC3812419

[B40] Wheeler M. J. Green D. J. Ellis K. A. Cerin E. Heinonen I. Naylor L. H. (2020). Distinct effects of acute exercise and breaks in sitting on working memory and executive function in older adults: a three-arm, randomised cross-over trial to evaluate the effects of exercise with and without breaks in sitting on cognition. Br. J. Sports Med. 54 (13), 776–781. 10.1136/bjsports-2018-100168 31036563

[B41] Wu C. C. Yang J. Wang X. Q. (2024). Analgesic effect of dance movement therapy: an fNIRS study. Neuroimage. 301, 120880. 10.1016/j.neuroimage.2024.120880 39362506

[B42] Xie H. Di X. Liu S. Zeng X. Meng Z. Xiao L. (2022). Tobacco use and cessation among college students - China, 2021. China CDC Wkly. 4 (21), 448–451. 10.46234/ccdcw2022.100 35686046 PMC9167610

[B43] Yao L. Sun G. Wang J. Hai Y. (2022). Effects of Baduanjin imagery and exercise on cognitive function in the elderly: a functional near-infrared spectroscopy study. Front. Public Health 10, 968642. 10.3389/fpubh.2022.968642 36249264 PMC9557749

[B44] Yu C. C. Muggleton N. G. Chen C. Y. Ko C. H. Liu S. (2021). The comparisons of inhibitory control and post-error behaviors between different types of athletes and physically inactive adults. PLoS One 16 (8), e0256272. 10.1371/journal.pone.0256272 34398917 PMC8366960

[B45] Zhang Y. Shi W. Wang H. Liu M. Tang D. (2021). The impact of acute exercise on implicit cognitive reappraisal in association with left dorsolateral prefronta activation: a fNIRS study. Behav. Brain Res. 406, 113233. 10.1016/j.bbr.2021.113233 33737088

